# Short version of the smartphone addiction scale: Measurement invariance across gender

**DOI:** 10.1371/journal.pone.0283256

**Published:** 2023-03-22

**Authors:** Heng Yue, Xiwen Yue, Bo Liu, Xueshan Li, Yaohua Dong, Hugejiletu Bao

**Affiliations:** 1 School of Psychology, Inner Mongolia Normal University, Hohhot, China; 2 Beidou College, Wuhan Qingchuan University, Wuhan, China; 3 College of Physical Education, Inner Mongolia Normal University, Hohhot, China; University of Concepcion Faculty of Medicine: Universidad de Concepcion Facultad de Medicina, CHILE

## Abstract

The Smartphone Addiction Scale Short Version (SAS-SV) has been widely used in research, but little is known about the measurement invariance across gender. The current study measured SAS-SV invariance between male and female college students in a sample of 1112 participants. Single- and multiple-group confirmatory factor analyses (CFAs) of smartphone addiction symptom ratings were conducted using R program with RStudio. SAS-SV was psychometrically robust in measuring the severity of smartphone addiction among college students, as well as the gender-based invariance. The differences in SAS-SV between male and female participants were likely to represent true gender differences, and meaningful comparisons could be made.

## 1. Introduction

Smartphones have led to dramatic changes in daily activities and behaviors. Applications allow communication with others, e-mail access, enjoyment of music/videos/films, game playing and schedule management. Smartphones may be beneficial, expanding horizons, promoting safety, alleviating stress, maintaining relationships and finding useful information [1–3] which has contributed to their indispensability [4]. However, improper smartphone use leads users towards unintentional time-wasting, and immoderate use carries the risk of smartphone addiction with an impact on physical and mental health.

Smartphone addiction describes a lack of control of smartphone use with negative consequences [5], and is considered a technological or behavioral addiction. Smartphone addicts may display the six core performances of addiction: salience, mood modification, tolerance, withdrawal, conflict and relapse [1, 6]. Smartphone addiction has been positively correlated with mental distress, such as depression, anxiety, loneliness, stress and boredom in empirical studies [7–10] and linked to poor sleep quality, impaired learning and acquisition and premature cognitive decline [11]. Adverse physical effects have also been reported, such as dry eye [12], musculoskeletal pain [13], hypertension [14], body dysfunction and weakened immunity [15]. These conditions are associated with a decrease in psychological well-being [16] and reduced life satisfaction [17]. Therefore, measures to prevent or treat this addictive behavior are needed, with the establishment of an accurate diagnostic criterion being the first step.

The Mobile Phone Addiction Index (MPAI) [18], Mobile Phone Problem Use Scale (MPPUS) [19], Problematic Mobile Phone Use Questionnaire [20], Smartphone Addiction Inventory (SPAI) [21], Smartphone Application-Based Addiction Scale (SABAS) [22] and Smartphone Addiction Scale (long version and short version) have all been introduced to assess the severity of smartphone addiction [23, 24] and the Smartphone Addiction Scale short version (SAS-SV) has been widely used. SAS-SV is considered simple, easy and efficient [23] with good reliability and validity in different cultural contexts. It has been translated into English [23], Turkish [25], Chinese [26], Italian [27], Iranian [28], Moroccan [29], Brazilian [30], Spanish and French [31]. An interesting outcome of SAS-SV-based research is the gender difference in smartphone addiction severity.

Gender differences were tested during SAS-SV development, but with controversial results. The mean scores of females were significantly higher than those of males [23], and females from Hong Kong were more likely to be addicted to smartphones than males [26]. Similar results were found among Japanese college students [32]. However, other studies have found higher scores for males than females and greater likelihood of addiction among the male population [30, 33, 34], and no gender difference has also been an outcome [35–38]. The last outcome indicated homogeneous symptoms among males and females and equivalent levels of smartphone addiction, although content viewed and motivation/justification might be varied.

The above controversy has arisen due to the assumption that “the measurement and the structure of the underlying construct are equivalent across groups” [39–41] and the failure to perform SAS-SV invariance tests. It remains unclear whether the functions of SAS-SV are identical across male and female participants, and thus, verification of the lack of cross-gender measurement bias is required. Measurement invariance of the SAS-SV across gender groups should be examined.

Measurement invariance refers to whether the same metric produces identical measurements under different conditions during observation and research [42]. For a given factorially defined construct, mathematical equality of corresponding measurement parameters, such as loadings and intercepts, across two or more groups would indicate measurement invariance [43]. Indeed, measurement invariance has been considered a prerequisite for the comparison of gender, age, culture and other dimensions [41]. The demonstration of measurement invariance across the cis genders would allow for the meaningful comparison of their mean scores [44]. Measurement invariance testing is often performed through an iterative process. A series of increasingly restrictive confirmatory factor analytic (CFA) models determine the extent to which measurement parameters (loadings and intercepts) are equivalent across different samples or time points [40, 45, 46]. Associations between manifest indicators and latent constructs, such as regression intercepts, factor loadings and residual (error/uniqueness) variances, are involved. In practice, measurement invariance includes four hierarchically nested parts: configural, metric, scalar and error variance invariance. Accordingly, the four aspects of SAS-SV should be tested to provide evidence for the gender-based measurement invariance of this scale.

Cross-culture and cross-time SAS-SV invariance have been investigated previously, and the measurement equivalence is verified [31, 47]. Cross-gender equivalence has been established for the revised Chinese Smartphone Addiction Scale (SAS-RC) [48]. However, this scale has been adapted from the Smartphone Addiction Scale long version [24] and lacks several items of the SAS-SV. University students engage in high levels of smartphone use [49], and addiction is prevalent in China [50, 51], as elsewhere. Therefore, the aim of the current study was to examine the cross-gender equivalence of SAS-SV in a population of Chinese university students.

## 2. Methods

### 2.1. Participants and procedure

Data about participants’ levels of smartphone addiction was collected by an anonymous online survey. A total of 1112 college students, 529 of whom were male and 583 female, with a mean age of 20.28 ± 1.43 years (range: 18–25), participated in the study.

Ethical approval was granted by the research ethics committee of the Inner Mongolia Normal University (College of Psychology). Verbal informed consent was given by all participants and their head teachers for the use of anonymized data for research and a link to the questionnaire sent via WeChat or QQ groups during participants’ spare time.

### 2.2. Measurements

SAS-SV is a self-reported measure for assessment of smartphone addiction severity. The scale consists of 10 items [23]. All participants were told that “please indicate to what extent you agree that this is true for you”, and were asked to answer the following question: “Is missing planned work due to smartphone use?”. Responses were recorded by a 6-point Likert scale ranging from 1 (strongly disagree) to 6 (strongly agree). Cronbach’s alpha for the scale across the whole group was 0.883, with 0.895 and 0.872 for male for female participants, respectively. The construct validity of SAS-SV has been verified in various cultures and countries [23, 25–31], and it is correlated with numerous measures [52, 53], thus providing convergent and concurrent construct evidence for the validity of this scale.

### 2.3. Statistical analysis

SPSS 25.0 was used for descriptive statistics, normality evaluation and reliability assessment. “lavaan” [54] and “semTools” [55] in R programming language with RStudio [56] were used for confirmatory factor analysis (CFA) and evaluation of configural, metric, scalar and error variance invariance across genders. Data normality was assessed by skewness and kurtosis. Item scores were regarded as normally distributed if the absolute values of the two indices did not exceed 2 [57]. Weighted least squares means and variance adjusted (WLSMV) estimator was used for single- and multiple-group CFAs [58]. Multiple indices were adopted to examine the goodness of fit of CFA models, including the significance of χ^2^, ratio between χ^2^ and degrees of freedom (df), root mean square error of approximation (RMSEA), comparative fit index (CFI) and standardized root mean square residual (SRMR). A good fit of the CFA model to the data was indicated by a non-significant χ^2^, χ^2^/df < 5, RMSEA < 0.08, CFI > 0.95, TLI > 0.95, SRMR < 0.08 [59–62].

Two single-group CFAs were conducted to assess the fit of the model in the two gender groups separately. Multiple-group CFA was then used to analyze SAV-SV measurement invariance across the two groups. Four hierarchically nested models were constructed and compared in an iterative manner. The raw score means were compared between groups to test whether males and females had an equivalent levels of smartphone addiction.

SAS-SV measurement invariance was assessed via the overall fit for each model and comparisons between nested models. Measurement invariance was supported if fit indices were acceptable and nested model comparisons fulfilled the following criteria: (1) RMSEA change (ΔRMSEA) < 0.050; (2) CFI change (ΔCFI) < 0.004; (3) SRMR change (ΔSRMR) < 0.010 [63, 64]. Chi-square change was not a criterion due to its sensitivity to sample size and limited capacity to distinguish invariant from non-invariant models [41, 44, 45, 65] and results are reported merely for completeness.

## 3. Results

### 3.1. Descriptive statistics

Descriptive statistics for all male and female participants are shown in Table 1. Means (standard deviations) ranged from 3.27 to 3.88 (1.403 to 1.480). The mean for males ranged from 3.18 to 3.86 (1.371 to 1.460) and for females from 3.32 to 3.90 (1.397 to 1.500). The absolute values for skewness and kurtosis in all three groups were all less than 2, indicating normal distribution of all 10 items [65].

**Table 1:** Descriptive statistics of the SAS-SV [total (male, female)].

Item	Mean	Standard Deviation	Skewness	Kurtosis
1	3.38 (3.37, 3.39)	1.415 (1.406, 1.424)	0.001 (0.045, -0.038)	-0.833 (-0.836, -0.825)
2	3.27 (3.18, 3.35)	1.417 (1.385, 1.441)	0.069 (0.142, -0.002)	-0.881 (-0.883, -0.865)
3	3.34 (3.36, 3.32)	1.480 (1.460, 1.500)	0.045 (0.038, 0.052)	-1.023 (-1.007, -1.038)
4	3.39 (3.41, 3.37)	1.441 (1.440, 1.443)	-0.001 (0.033, -0.033)	-0.919 (-0.901, -0.937)
5	3.88 (3.86, 3.90)	1.458 (1.441, 1.475)	-0.315 (-0.299, -0.331)	-0.828 (-0.837, -0.817)
6	3.51 (3.47, 3.54)	1.456 (1.448, 1.462)	-0.011 (0.040, -0.057)	-0.951 (-0.916, -0.973)
7	3.54 (3.47, 3.61)	1.403 (1.407, 1.397)	-0.026 (0.123, -0.162)	-0.815 (-0.833, -0.745)
8	3.45 (3.43, 3.47)	1.462 (1.454, 1.470)	0.026 (0.091, -0.032)	-0.944 (-0.943, -0.938)
9	3.49 (3.44, 3.53)	1.434 (1.445, 1.425)	0.000 (0.059, -0.054)	-0.889 (-0.881, -0.885)
10	3.59 (3.53, 3.64)	1.406 (1.371, 1.437)	-0.082 (-0.022, -0.139)	-0.870 (-0.777, -0.932)

### 3.2. Single-group confirmatory factor analysis

Single-group CFAs for the whole group and for male and female participants are presented in Tables 2 and 3. SAS-SV yielded adequate model fit statistics of a significant χ^2^, χ^2^/df = 5.227, RMSEA = 0.062 CFI = 0.985, TLI = 0.980, SRMR = 0.029 with standardized factor loadings ranging from 0.581 to 0.782 for the whole sample. Although χ^2^ was significant. the value of χ^2^/df was slightly higher than the recommended cutoff value [60]. As the chi-square statistic was sensitive to sample size [41, 65] and all other model fit indices were within acceptable ranges, the model was considered to be appropriate.

**Table 2:** Results of single-group confirmatory factor analysis (model fit indices).

Sample	χ^2^	df	χ^2^/df	RMSEA	CFI	TLI	SRMR
All participants (n = 1112)	182.941**	35	5.227	0.062	0.985	0.980	0.029
Male (n = 529)	127.253**	35	3.634	0.071	0.983	0.978	0.032
Female (n = 583)	114.203**	35	3.263	0.062	0.982	0.977	0.034

Note

** *p*<0.01

**Table 3:** Results of single-group confirmatory factor analysis (standardized factor loadings).

Sample	Item1	Item2	Item3	Item4	Item5	Item6	Item7	Item8	Item9	Item10
All participants (n = 1112)	0.581	0.661	0.670	0.658	0.638	0.706	0.747	0.718	0.725	0.782
Male (n = 529)	0.586	0.687	0.701	0.676	0.671	0.716	0.802	0.739	0.756	0.775
Female (n = 583)	0.575	0.643	0.645	0.642	0.607	0.699	0.696	0.701	0.695	0.792

In order to test whether the unidimensional model is the best fitting model, according to the suggestions of Ronald F. Levant and his colleagues [66], variance composition of the SAS-SV was assessed based on the common factors, hierarchical, bifactor model. However, none of these models could be identified. Therefore, the unidimensional model of SAS-SV was considered acceptable and regarded as the best fitting model for this scale.

Fit indices indicated good data fitting to the model for the male sample with a significant χ^2^, χ^2^/df = 3.634, RMSEA = 0.071, CFI = 0.983, TLI = 0.978, SRMR = 0.032 and standardized factor loadings ranging from 0.586 to 0.802 and for the female sample with a significant χ^2^, χ^2^/df = 3.263, RMSEA = 0.062, CFI = 0.982, TLI = 0.977, SRMR = 0.034 and standardized factor loadings ranging from 0.575 to 0.792. According to the criteria mentioned above, the unidimensional model of SAS-SV was considered acceptable for both male and female groups.

### 3.3. Measurement invariance across gender

Multiple-group CFAs are presented in Table 4.

**Table 4:** Model fit indices for measurement invariance tests.

	Model Fit Indices						Model Difference					
Model	χ^2^	df	χ^2^/df	RMSEA	CFI	SRMR	ΔM	Δχ^2^	Δdf	ΔRMSEA	ΔCFI	ΔSRMR
M1	239.812	70	3.426	0.066	0.983	0.033	-	-	-	-	-	-
M2	276.192	109	2.534	0.053	0.983	0.033	M2 VS. M1	36.380	39	-0.013	0.000	0.000
M3	270.023	118	2.288	0.048	0.985	0.033	M3 VS. M2	-6.169	9	-0.005	0.002	0.000
M4	310.377	128	2.425	0.051	0.981	0.036	M4 VS. M3	40.354	10	0.003	-0.004	0.003

M1: configural invariance; M2: metric invariance; M3: scalar invariance; M4: strict invariance.

The configural invariance model (M1) was estimated without applying any equality constraint, and the model fit indices suggested good fitting of the data to the model (χ^2^/df = 3.426, RMSEA = 0.066, CFI = 0.983, SRMR = 0.033). Thus, configural invariance was supported.

The metric invariance model (M2) was tested by restricting factor loadings to be equivalent between the two gender groups. The model fit the data well (χ^2^/df = 2.534, RMSEA = 0.053, CFI = 0.983, SRMR = 0.033). ΔRMSEA was 0.013, ΔCFI 0.000 and ΔSRMR 0.000 between M1 and M2 which were all within the recommended guidelines. Thus, metric invariance of SAS-SV across gender was supported.

The scalar invariance model (M3) was assessed by constraining factor loadings and item intercepts to be identical across the two gender groups and good fitting of the data to the model was shown (χ^2^/df = 2.288, RMSEA = 0.048, CFI = 0.985, SRMR = 0.033). ΔRMSEA was 0.005, ΔCFI 0.002 and ΔSRMR 0.000 between M2 and M3 which were all within the recommended criteria. Therefore, SAS-SV scalar invariance across gender was confirmed.

Strict invariance of the scale (M4) was assessed by holding factor loadings, intercepts and residual item variance equal across male and female groups and good fitting of the data to the model was shown (χ^2^/df = 2.425, RMSEA = 0.051, CFI = 0.981, SRMR = 0.036). ΔRMSEA was 0.003, ΔCFI 0.004 and ΔSRMR 0.003 compared with M3, which were all within the recommended criteria. Therefore, strict SAS-SV invariance across gender was demonstrated.

Lastly, the raw score means were compared between the two groups. The results of the independent t test were: t (1110) = -1.01, p = 0.31, indicating that males and females had an equivalent level of smartphone addiction.

## 4. Discussion

The SAS-SV has been frequently used to assess the severity of smartphone addiction but without full characterization of cross-gender equivalence. The current study tested SAS-SV measurement invariance among male and female participants.

Single-group CFA indicated acceptable data fitting for the unidimensional model in the whole group and in both gender sub-groups, consistent with previous Chinese language and other studies [25–31]. However, the previous Chinese study was restricted to Hong Kong residents [26] and may be subject to some cultural differences from mainland China. Therefore, testing of SAS-SV factor structure in the mainland Chinese population was required. The present study suggests that this scale is a robust and useful tool for both male and female residents of mainland China. Scalar invariance is regarded as an imperative procedure for comparing latent means, which conforms to the identical operational definition, including equal intervals and zero points across the indicators between the two gender groups [67]. Thus, intercepts of items of the scale were equivalent across gender. In addition, multiple-group CFA demonstrated SAS-SV strict invariance, indicating homogeneous residual variances of manifest indicators (items) across male and female university students [68]. Thus, an observed variable (item) may be predicted from the underlying construct (smartphone addiction) with an identical degree of measurement error across gender [44]. Moreover, raw score means of the two gender groups were compared, and the results indicated that males and females had an equivalent level of smartphone addiction.

SAS-SV’s unidimensional structure was found to be equivalent for male and female university students. The configural, metric, scalar and strict invariance measurement invariance tests also demonstrated the gender invariance of the psychometric features. Identical SAS-SV scores from the two gender groups represented equal levels of smartphone addiction in male and female students. Any gender differences might reflect the true difference in the severity of smartphone addiction, but not measurement bias. Therefore, the SAS-SV may be used with confidence to measure and compare the degree of smartphone addiction across gender in Chinese university student samples. Previous research has identified different content preferences between male and female smartphone users, with males preferring games apps and females preferring social media apps [69], However, the current study suggests no significant gender differences in addictive symptoms. SAS-SV items were interpreted in a comparable way by male and female university students and the scale was robust. The current results support the usage of SAS-SV, free from measurement bias, in research and clinical contexts.

We acknowledge several limitations to the current study. First, only measurement invariance across two self-identified genders (male and female) was evaluated, and non-binary and transgender individuals were not taken into account, giving an incomplete view according to Ronald F. Levant and his colleagues [70]. Therefore, future studies are needed to investigate SAS-SV measurement invariance across different gender identities. Second, only college student samples were included in this study, and smartphone addiction is also prevalent in other populations, such as children, adolescents and adults. Thus, gender invariance of the scale should also be investigated in different sample populations. Third, the current study was conducted on a convenience sample, which could reduce the generalizability of the results. Future studies with different sampling methods are warranted.

## 5. Conclusions

The structural validity and measurement equivalence of the SAS-SV was assessed by single- and multiple-group CFAs. Empirical evidence is presented to support the unidimensional structure and full measurement equivalence of the SAS-SV across gender. Differences in SAS-SV scores between male and female college students indicated obvious gender differences in smartphone addiction, which were not due to measurement deviation of the scale items. The current study contributes to meaningful interpretations of gender comparisons of smartphone addiction. There are practical implications for researchers, clinicians and the general public.
